# Mesenchymal stem cells pretreated with interferon-gamma attenuate renal fibrosis by enhancing regulatory T cell induction

**DOI:** 10.1038/s41598-024-60928-4

**Published:** 2024-05-04

**Authors:** So Kurawaki, Ayumu Nakashima, Naoki Ishiuchi, Ryo Kanai, Satoshi Maeda, Kensuke Sasaki, Takao Masaki

**Affiliations:** 1 Department of Nephrology, Hiroshima University Hospital, 1-2-3 Kasumi, Minami-ku, Hiroshima, 734-8551 Japan; 2 Department of Stem Cell Biology and Medicine, Graduate School of Biomedical & Health Sciences, Hiroshima University, 1-2-3 Kasumi, Minami-ku, Hiroshima, 734-8553 Japan; 3 TWOCELLS Company, Limited, 16-35 Hijiyama-honmachi, Minami-ku, Hiroshima, 732-0816 Japan

**Keywords:** Mesenchymal stem cells, Kidney, Cell biology

## Abstract

Mesenchymal stem cells (MSCs) exert their anti-inflammatory and anti-fibrotic effects by secreting various humoral factors. Interferon-gamma (IFN-γ) can enhance these effects of MSCs, and enhancement of regulatory T (Treg) cell induction is thought to be an underlying mechanism. However, the extent to which Treg cell induction by MSCs pretreated with IFN-γ (IFN-γ MSCs) ameliorates renal fibrosis remains unknown. In this study, we investigated the effects of Treg cell induction by IFN-γ MSCs on renal inflammation and fibrosis using an siRNA knockdown system. Administration of IFN-γ MSCs induced Treg cells and inhibited infiltration of inflammatory cells in ischemia reperfusion injury (IRI) rats more drastically than control MSCs without IFN-γ pretreatment. In addition, administration of IFN-γ MSCs more significantly attenuated renal fibrosis compared with control MSCs. Indoleamine 2,3-dioxygenase (IDO) expression levels in conditioned medium from MSCs were enhanced by IFN-γ pretreatment. Moreover, IDO1 knockdown in IFN-γ MSCs reduced their anti-inflammatory and anti-fibrotic effects in IRI rats by reducing Treg cell induction. Our findings suggest that the increase of Treg cells induced by enhanced secretion of IDO by IFN-γ MSCs played a pivotal role in their anti-fibrotic effects. Administration of IFN-γ MSCs may potentially be a useful therapy to prevent renal fibrosis progression.

## Introduction

The prevalence of acute kidney injury (AKI) has been increasing in recent decades and is recognized as a worldwide health problem^1,2^. AKI is associated with subsequent development of chronic kidney disease (CKD)^3^. Therefore, mechanisms underlying AKI-to-CKD progression have been extensively investigated over the past decade. The main pathological feature of AKI-to-CKD progression is interstitial fibrosis, which can result from one or more pathological mechanisms, such as hypoxia^4^, endothelial dysfunction, microvascular rarefaction^5^, inflammation^6^, transforming growth factor (TGF)-β1 production^7^, and epithelial-mesenchymal transition (EMT)^8^. Notably, among these mechanisms, inflammation plays a major role in fibrosis progression^9^.

Numerous studies have reported that sustained pro-inflammatory stimuli activate fibroblasts, resulting in fibrosis^10,11^. In recent years, regulatory T (Treg) cells have received much attention for their role as an inhibitory system against this progressive inflammation^12,13^. Treg cells can reportedly suppress excessive immune responses by various mechanisms, including direct cell–cell contact^14^, depletion of interleukin (IL)-2^15,16^, and release of soluble inhibitory factors like IL-10^17,18^. In addition, Treg cells exert protective effects against renal injury^19^. Therefore, increasing Treg cell induction within the injury site is potentially a novel therapeutic strategy for renal fibrosis.

Mesenchymal stem cells (MSCs) are multipotent adult stem cells^20^ isolated from various tissues, such as bone marrow, adipose tissue, and umbilical cord^21^. We elucidated that the anti-fibrotic effects of xenogeneic and allogeneic MSCs are almost equal^22^. Moreover, many studies have revealed that xeno therapy using human MSCs is effective for mouse models of renal injury^23,24^. MSCs contribute to tissue regeneration by secreting various soluble factors^25,26^. MSCs are known to favor the generation of Treg cells, producing anti-inflammatory effects^27^.

We previously demonstrated that MSCs pretreated with IFN-γ (IFN-γ MSCs) potently ameliorate renal fibrosis. Several studies have demonstrated that pretreatment with interferon-gamma (IFN-γ) promotes Treg cell induction by enhancing indoleamine 2,3-dioxygenase (IDO) secretion from MSCs^28,29^. These findings led us to hypothesize that IFN-γ MSCs attenuate renal fibrosis by enhancing regulatory T cell induction.

In this study, we investigated the effects of Treg cell induction by IFN-γ MSCs on renal inflammation and fibrosis in a unilateral renal ischemia reperfusion injury (IRI) rat model with contralateral nephrectomy using a siRNA knockdown system.

## Results

### MSCs pretreated with IFN-γ inhibited inflammatory cell infiltration by inducing regulatory T cells

**Figure 1:**
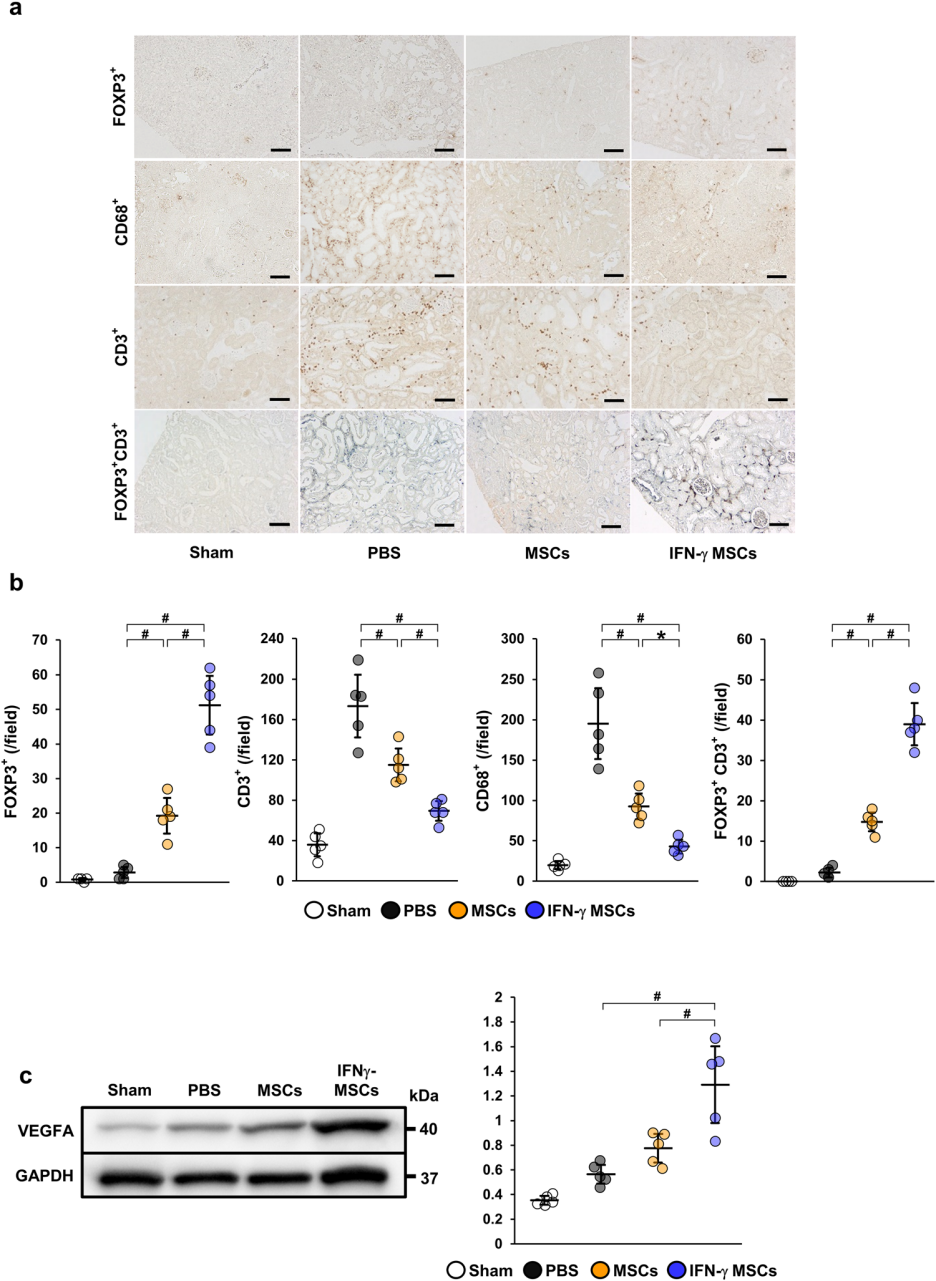
Anti-inflammatory effects of mesenchymal stem cells (MSCs) by regulatory T (Treg) cells induction in the kidney of ischemia reperfusion injury (IRI) rats. MSCs pretreated with IFN-γ (IFN-γ MSCs) or MSCs untreated were injected immediately after IRI induction. Infiltration of T cells and macrophages into injured kidney of MSC-injected IRI rats was assessed at day 7 post-IRI by immunostaining. (**a**) Representative image of immunohistochemical staining of FOXP3, CD3, and CD68 and double immunostaining of FOXP3 (brown) and CD3 (blue) in kidney sections (scale bar = 100 µm). (**b**) Quantification of FOXP3-, CD3-, CD68-positive cells and double-positive (FOXP3 and CD3) cells (n = 5 in each group). (**c**) Western blot analysis of VEGFA in the kidney cortex of IRI rats. Graphs show densitometric analyses of VEGFA expression levels normalized to the GAPDH expression level (n = 5 in each group). Full-length blots are presented in Supplementary Fig. 1a. Data indicate mean ± S.D. ^#^*P* < 0.01, *P < 0.05 (one-way ANOVA followed by Bonferroni’s post-hoc test).

We first investigated whether IFN-γ MSCs had a stronger immunosuppressive effect than untreated MSCs in an experimental IRI model. After the IRI procedure, 5 × 10^5^ cells untreated MSCs (control MSCs), IFN-γ MSCs, or PBS was injected into rats through the abdominal aorta. At 7 days after IRI, immunohistochemical staining showed that numbers of cells positive for FOXP3, a Treg marker, were not drastically changed in IRI rats injected with PBS compared with the findings in the control group. Meanwhile, numbers of FOXP3-positive cells were increased in IRI rats injected with control MSCs and further upregulated in IRI rats injected with IFN-γ MSCs (Fig. 1a,b). In contrast, numbers of cells positive for CD3 (a pan T cell marker) and CD68 (a macrophage marker) were increased in the PBS group. These observations of increased expression were suppressed in the control MSCs group, and further suppressed in the IFN-γ MSCs group (Fig. 1a,b). We also performed double immunostaining for FOXP3 and CD3 to identify Treg cells. Positive cells for both FOXP3 and CD3 were increased in the MSCs group, and a further increase was observed in the IFN-γ MSCs group (Fig. 1a,b).

### MSCs pretreated with IFN-γ increased vascular endothelial growth factor in IRI rats

Upregulation of vascular endothelial growth factor (VEGF) in IRI rats is critical to prevent AKI progression. Therefore, we investigated the effects of IFN-γ MSCs on VEGFA expression in IRI rats at 7 days post-injection. The protein level of VEGFA was upregulated in the control MSCs group and further upregulation was observed in the IFN-γ MSCs group (Fig. 1c, Additional File 1A: Fig. S1a).

### MSCs pretreated with IFN-γ suppressed IRI-induced tubulointerstitial fibrosis and extracellular matrix accumulation

**Figure 2:**
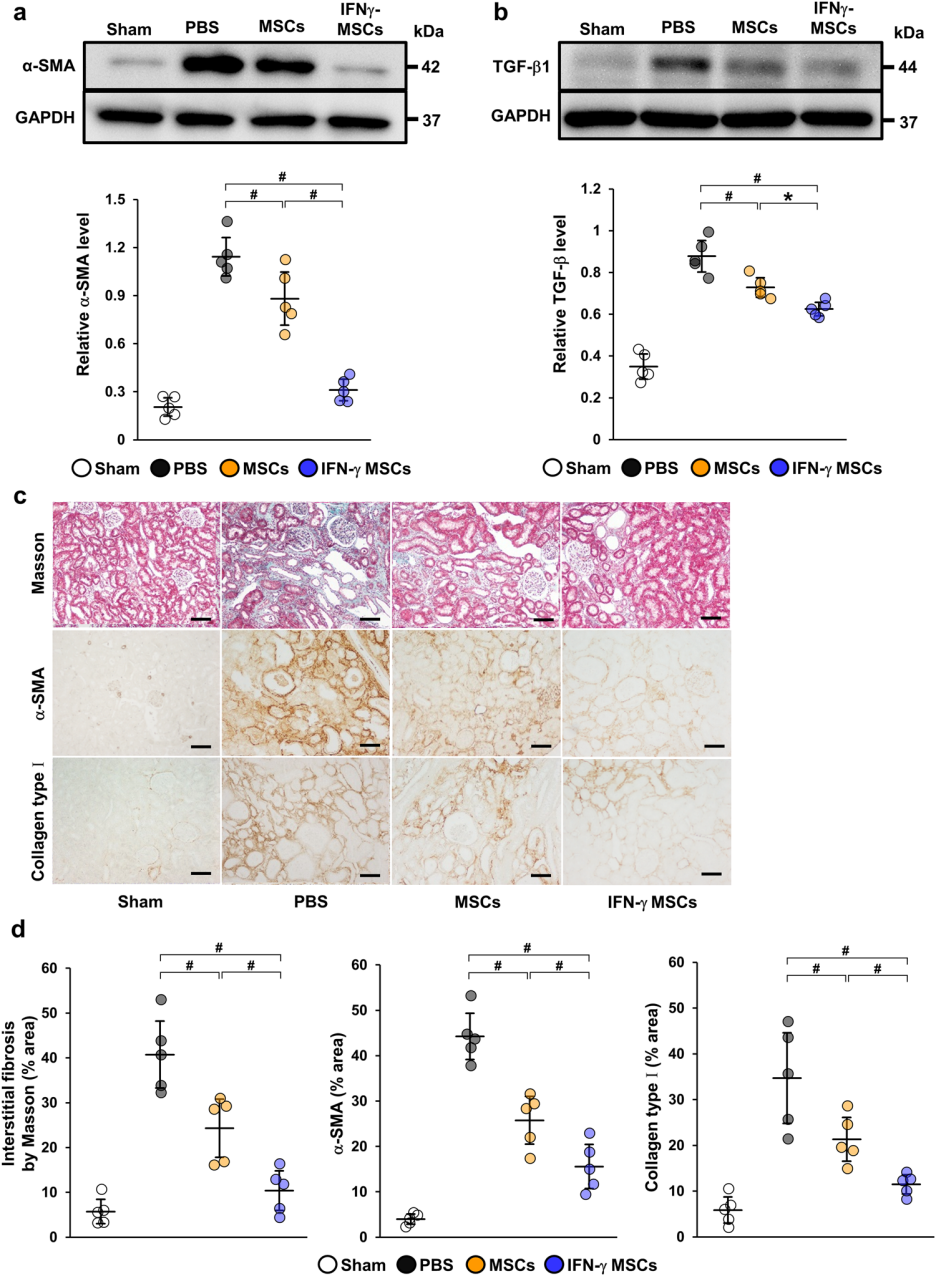
Anti-fibrotic effects of IFN-γ MSCs in the kidney of IRI rats. Protein levels of fibrosis markers were evaluated at day 21 post-IRI by western blot (**a**, **b**), Masson’s trichrome staining and immunohistochemical (**c**) analyses. (**a**, **b**) Western blot analysis of α-SMA and TGF-β1 in the kidney cortex of IRI rats. Graphs show densitometric analyses of α-SMA and TGF-β1 expression levels normalized to the GAPDH expression level (n = 5 in each group). Full-length blots are presented in Supplementary Fig. 1b, 1c. (**c**) Representative images of Masson’s trichrome staining and immunohistochemical staining of α-SMA and collagen type I in kidney sections (scale bar = 100 µm). (**d**) Quantification of interstitial fibrosis area and α-SMA- and collagen type I-positive areas as percentages of the total area (n = 5 in each group). Data indicate mean ± S.D. ^#^*P* < 0.01, *P < 0.05 (one-way ANOVA followed by Bonferroni’s post-hoc test).

Next, we investigated the effect of IFN-γ MSCs on tubulointerstitial fibrosis in IRI rats at 21 days post-injection. We found that protein levels of α-smooth muscle actin (α-SMA) and TGF-β1 were remarkably increased in the PBS group, suppressed in the control MSCs group, and further suppressed in the IFN-γ MSCs group (Fig. 2a,b, Additional File 1A: Fig. S1b,c). Masson’s trichrome staining revealed that the extent (area) of interstitial fibrosis was more significantly suppressed in the IFN-γ MSCs group compared with that in the control MSCs group. Similarly, α-SMA- and collagen type I-positive areas were more significantly reduced in the IFN-γ MSCs group compared with those in the control MSCs group (Fig. 2c,d).

### IFN-γ pretreatment increased indoleamine 2,3-dioxygenase expression in conditioned medium from MSCs

**Figure 3:**
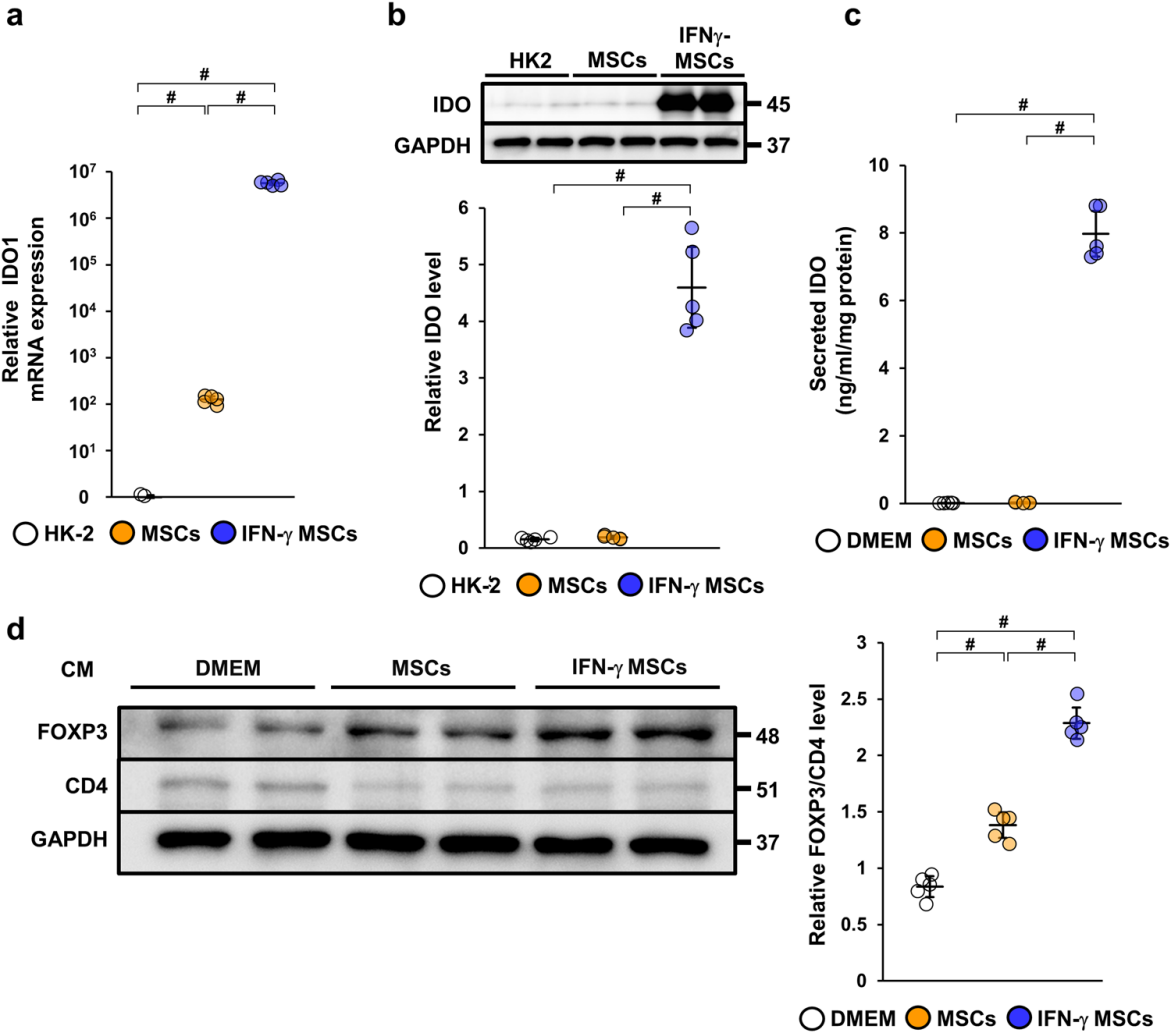
Effects of conditioned medium obtained from IFN-γ MSCs on differentiation of naïve CD4 T cells into regulatory T cells. mRNA and protein levels of indoleamine 2,3-dioxygenase (IDO) were evaluated by quantitative real-time polymerase chain reaction (**a**) and western blot (**b**) analyses. (**a**) Expression levels of IDO1 mRNA in HK-2 cells, MSCs, and IFN-γ MSCs (n = 5 in each group). (**b**) Western blot analysis of IDO in HK-2 cells, MSCs, and IFN-γ MSCs. Graph shows densitometric analysis of IDO expression levels normalized to the GAPDH expression level (n = 5 in each group). Full-length blots are presented in Supplementary Fig. 1d. (**c**) Concentration of IDO in conditioned medium from MSCs was evaluated by an ELISA analysis at 48 h after IFN-γ stimulation. DMEM containing 0.1% FBS was used as a negative control. Graph shows IDO concentrations in DMEM containing 0.1% FBS or conditioned medium from MSCs or IFN-γ MSCs (n = 5 in each group). (**d**) Differentiation of naïve CD4 T cells into regulatory T cells were evaluated by FOXP3 and CD4 protein levels. Naïve CD4 T cells were isolated from human peripheral blood mononuclear cells and were incubated with DMEM containing 0.1% FBS or conditioned medium obtained from MSCs or IFN-γ MSCs. Five days later, the cells were harvested and subjected to western blot analysis of FOXP3 and CD4. Graph shows densitometric analysis of FOXP3/CD4 expression levels normalized to the GAPDH expression level (n = 5 in each group). Full-length blots are presented in Supplementary Fig. 1e. Data indicate mean ± S.D. ^#^*P* < 0.01 (one-way ANOVA followed by Bonferroni’s post-hoc test).

Previous studies demonstrated that IDO expression plays a direct role in inducing the conversion of naïve CD4 T cells into Treg cells^30,31^. Therefore, we examined IDO expression levels in MSCs and CM collected from MSCs. We found that mRNA and protein levels of IDO were more significantly upregulated in IFN-γ MSCs compared with those in control MSCs (Fig. 3a,b, Additional File 1B: Fig. S1d). Additionally, ELISA results show that the concentration of IDO in IFN-γ MSCs-CM was higher than in control MSCs-CM (Fig. 3c).

### Conditioned medium from IFN-γ MSCs enhanced differentiation of naïve CD4 T cells into FOXP3-positive regulatory T cells

To investigate whether IFN-γ MSCs mediated the induction of FOXP3-positive Treg cells, we isolated naïve CD4 T cells from PBMCs and cultured them with control MSCs-CM or IFN-γ MSCs-CM. IFN-γ MSCs-CM more significantly increased FOXP3 protein levels compared with control MSCs-CM (Fig. 3d, Additional File 1B: Fig. S1e).

### IDO1 knockdown in IFN-γ MSCs weakened their immunosuppressive effect on regulatory T cell induction

**Figure 4:**
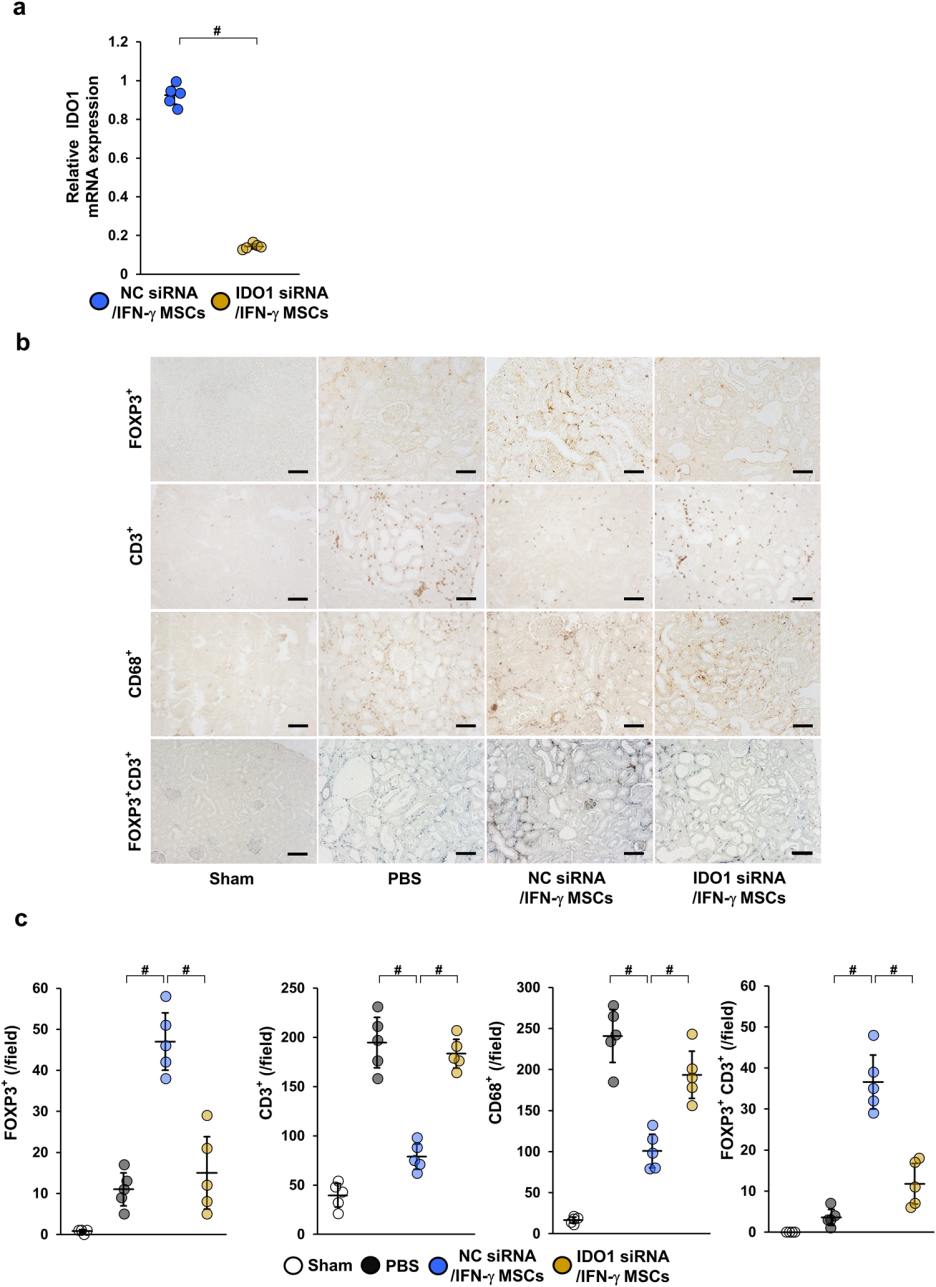
Suppressive effects of IDO1 siRNA on anti-inflammatory effects of IFN-γ MSCs in IRI rats. After MSCs transfected with NC siRNA or IDO1 siRNA were pretreated with IFN-γ, the cells were injected into rats immediately after IRI induction. Infiltration of T cells and macrophages into injured kidney of MSC-injected IRI rats were assessed at day 7 post-IRI by immunostaining. (**a**) Expression levels of IDO1 mRNA in IFN-γ-MSCs (n = 5 in each group). IFN-γ MSCs were transfected with IDO1 siRNA (IDO1 siRNA/IFN-γ MSCs) or negative control siRNA (NC siRNA/IFN-γ MSCs). (**b**) Representative images of immunohistochemical staining of FOXP3, CD3, and CD68 and double immunostaining of FOXP3 (brown) and CD3 (blue) in kidney sections at day 7 post-IRI (scale bar = 100 µm). (**c**) Quantification of FOXP3-, CD3-, CD68-positive cells and double-positive (FOXP3 and CD3) cells (n = 5 in each group). Data indicate mean ± S.D. ^#^*P* < 0.01 (one-way ANOVA followed by Bonferroni’s post-hoc test).

Next, to determine whether IDO secreted from IFN-γ MSCs was related to IFN-γ MSCs-mediated suppression of inflammation, we prepared IFN-γ MSCs transfected with IDO1 siRNA or negative control siRNA. First, we confirmed successful knockdown of IDO1 in siRNA-transfected IFN-γ MSCs (Fig. 4a). Next, we administered IRI rats with PBS or 5 × 10^5^ IFN-γ MSCs transfected with IDO1 siRNA (IDO1 siRNA/IFN-γ MSCs group) or negative control siRNA (NC siRNA/IFN-γ MSCs group). As shown in Fig. 4b,c, numbers of FOXP3-positive cells were increased in the NC siRNA/IFN-γ MSCs group. However, this effect totally disappeared in the IDO1 siRNA/IFN-γ MSCs group (Fig. 4b,c). Conversely, CD3- and CD68-positive cells were decreased in the NC siRNA/IFN-γ MSCs group and significantly increased in the IDO1 siRNA/IFN-γ MSCs group (Fig. 4b,c). In addition, positive cells for both FOXP3 and CD3 were increased in the NC siRNA/IFN-γ MSCs group, whereas this effect was reduced in the IDO1 siRNA/IFN-γ MSCs group (Fig. 4b,c).

### IDO1 knockdown in IFN-γ MSCs weakened their anti-fibrotic effects in IRI rats

**Figure 5:**
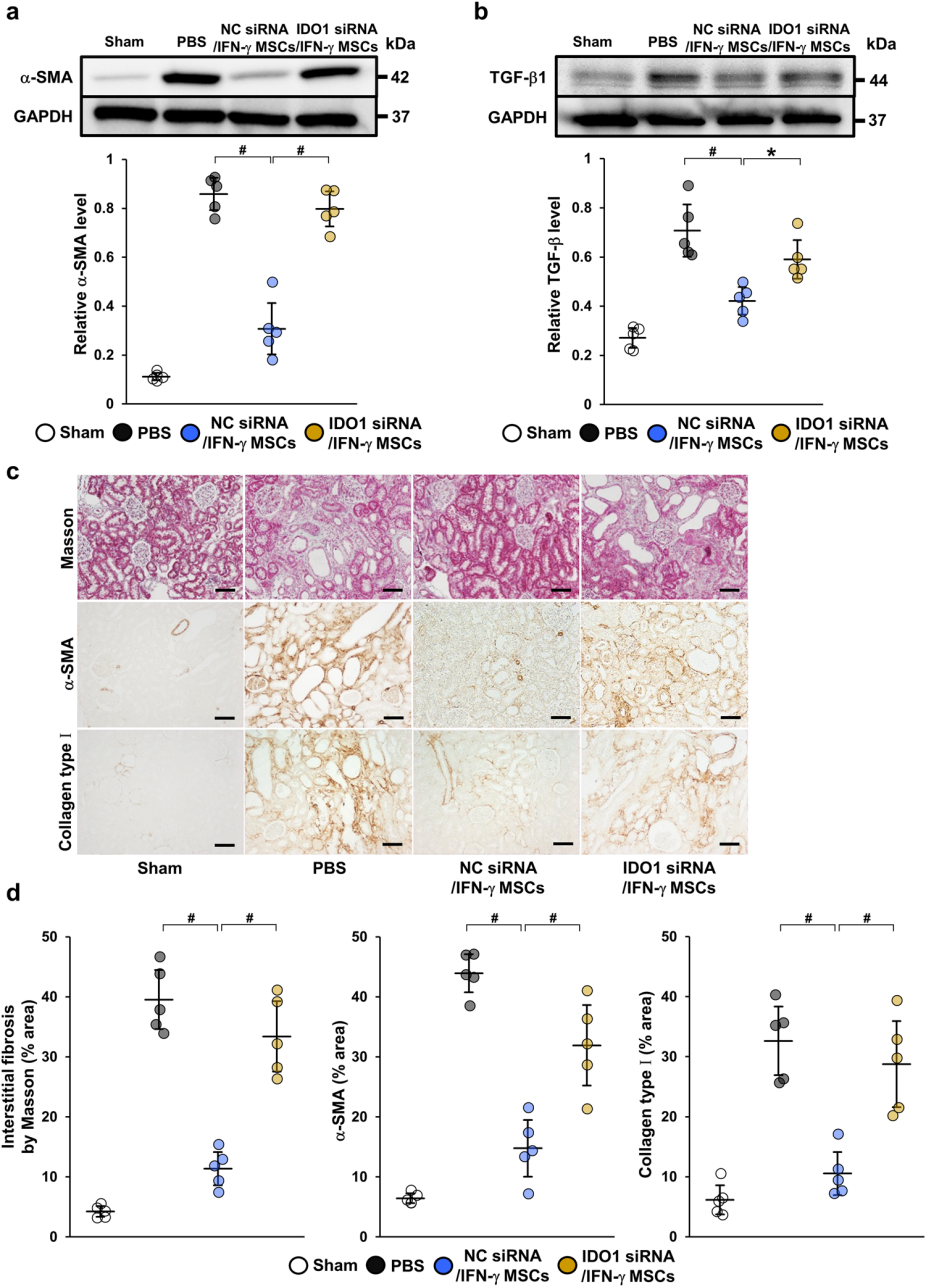
Inhibitory effects of IDO1 siRNA on anti-fibrotic effects of IFN-γ MSCs in IRI rats. After MSCs transfected with NC siRNA or IDO1 siRNA were pretreated with IFN-γ, the cells were injected into rats immediately after IRI induction. Protein levels of fibrosis markers were evaluated at day 21 post-IRI by western blot (**a**, **b**), Masson’s trichrome staining and immunohistochemical (**c**) analyses. (**a**, **b**) Western blot analysis of α-SMA and TGF-β1 in the kidney cortex of IRI rats. Graphs show densitometric analyses of α-SMA and TGF-β1 expression levels normalized to the GAPDH expression level (n = 5 in each group). Full-length blots are presented in Supplementary Fig. 1f, 1g. (**c**) Representative images of Masson’s trichrome staining and immunohistochemical staining of α-SMA and collagen type I of kidney sections (scale bar = 100 µm). (**d**) Quantification of interstitial fibrosis area and α-SMA- and collagen type I-positive areas as percentages of the total area (n = 5 in each group). Data indicate mean ± S.D. ^#^*P* < 0.01, *P < 0.05 (one-way ANOVA followed by Bonferroni’s post-hoc test).

Finally, we investigated the extent to which Treg cell induction by IFN-γ MSCs ameliorated renal fibrosis. Western blotting revealed that protein levels of α-SMA and TGF-β1 were increased in the PBS group and significantly ameliorated by injection of NC siRNA/IFN-γ MSCs, while these reductions were completely abrogated by injection of IDO1 siRNA/IFN-γ MSCs (Fig. 5a,b, Additional File 1C: Fig. S1f,g). Similarly, immunostaining showed that α-SMA- and collagen type I-positive areas were expanded in the PBS group and markedly ameliorated by injection of NC siRNA/IFN-γ MSCs, whereas these improvements were abolished by injection of IDO1 siRNA/IFN-γ MSCs (Fig. 5c,d).

## Discussion

The present study clarified that administration of IFN-γ MSCs induced Treg cells and ameliorated inflammation and tubulointerstitial fibrosis in IRI rats more drastically than unstimulated MSCs. Mechanistically, IFN-γ MSCs enhanced IDO secretion, while IFN-γ MSCs-CM strongly induced conversion of naïve CD4 T cells into Treg cells. In addition, IDO1 knockdown in IFN-γ MSCs diminished numbers of FOXP3-positive cells and reduced the therapeutic effect of IFN-γ MSCs on renal inflammation and fibrosis in IRI rats.

Advances in MSC biology have confirmed that sufficient inflammatory signals are required to activate the immunosuppressive properties of MSCs^32,33^. IFN-γ, a cytokine expressed in activated lymphocytes, reportedly modifies the immunomodulatory function of MSCs^34,35^. As a mechanism by which MSCs exert an anti-inflammatory effect, we previously showed that IFN-γ MSCs increase prostaglandin E2 (PGE2) secretion to induce immunosuppressive M2 macrophage polarization, leading to enhanced anti-inflammatory and anti-fibrotic effects^36^. The present study demonstrates that IFN-γ MSCs enhanced IDO secretion, thereby inhibiting infiltration of inflammatory cells by inducing Treg cells, leading to pronounced anti-fibrotic effects. Taken together, our findings indicate that IFN-γ MSCs enhanced the suppression of inflammatory cell infiltration through two pathways: induction of M2 macrophages by increasing PGE2 secretion and induction of Treg cells by upregulating IDO secretion.

MSCs exert immunosuppressive and therapeutic effects by a Treg cell-induced mechanism in various diseases models, such as transplantation^37,38^, autoimmune disorders^39,40^, and systemic inflammatory diseases^41,42^. MSCs remain dormant under normal conditions and exert anti-inflammatory effects when activated by cytokines released from immune cells in damaged tissues. This activation may require several days and some MSCs may be unable to change to the active form^36^, so normal MSCs may not exert sufficient therapeutic effects. In fact, our current study also revealed that control MSCs mediate only a few Treg cell induction in in vitro experiments and exert only slight anti-fibrotic efficacy in IRI rats. Therefore, we believe that pretreatment with IFN-γ is important when performing MSC therapy. Although IFN-γ MSCs reportedly increase Treg cell induction in in vitro^28,43^, the extent to which induction of Treg cells by IFN-γ MSCs ameliorates renal injury remains unknown. In this study, we observed that downregulation of MSC-induced Treg cells worsened renal inflammation and fibrosis in IRI rats injected with IFN-γ MSCs. Therefore, induction of Treg cells by IFN-γ MSCs was a central contributor to the suppression of renal fibrosis.

The biologic function of the IDO pathway is both counter-regulatory (controlling inflammation) and tolerogenic (creating acquired antigen-specific tolerance in T cells)^44^. IDO helps create a tolerogenic milieu by directly suppressing T cells and enhancing Treg-mediated immunosuppression^45^. In fact, in our experiments, IDO1 knockdown in MSCs pretreated with IFN-γ reduced Treg cell induction and attenuated their anti-inflammatory effects. An, et al. reported that PGE2 secreted from MSCs is involved in Treg cell induction^46^. We previously demonstrated that pretreatment with IFN-γ promoted PGE2 secretion from MSCs. Therefore, IFN-γ MSCs likely enhanced induction of Treg cells by enhancing secretion of both IDO and PGE2.

## Methods

### Cell culture

Human MSCs from bone marrow were purchased from Riken BioResource Research Center (Ibaraki, Japan) and cultured in Dulbecco’s Modified Eagle’s Medium (DMEM: Sigma-Aldrich, St. Louis, MO, USA) with 10% fetal bovine serum (FBS, Sigma-Aldrich). Cells were passaged four to five times before use for transplantation. HK-2 cells, a human proximal tubular cell line, were obtained from the American Type Culture Collection (Manassas, VA). These cells were cultured as described previously^47^.

### MSCs pretreatment with IFN-γ

MSCs were pretreated with or without recombinant human IFN-γ (PeproTech, Cranbury, NJ, USA) by the following method. When MSCs reached 70% confluence, IFN-γ was added to the medium to achieve a final concentration of 10 ng/mL. After 48 h, cells were collected and subjected to in vivo and in vitro analyses.

### Experimental animal model

Male Sprague Dawley (SD) rats (8 weeks old) were purchased from Charles River Laboratories Japan (Yokohama, Japan). Experimental procedures were approved by the Institutional Animal Care and Use Committee of Hiroshima University (Hiroshima, Japan) (Permit Nos. A15-66 and A17-75) and conducted in accordance with the Guide for the Care and Use of Laboratory Animals, 8th ed, 2010 (National Institutes of Health, Bethesda, MD, USA). This study is reported in accordance with ARRIVE guidelines. To establish the animal model, SD rats were randomly divided in 6 groups (n = 5 in each group): sham, PBS (control), MSCs, IFN-γ MSCs, NC siRNA/IFN-γ MSCs and IDO1 siRNA/IFN-γ MSCs groups. All procedures were performed under anesthesia with injection of agents composed of midazolam, medetomidine, and butorphanol. Right nephrectomy was performed 7 days prior to IRI of the left kidney. Renal IRI was induced by transiently clamping the unilateral renal artery. After a laparotomy was performed, the left kidney was exposed. Next, the renal pedicle was clamped by atraumatic vascular clamps for 45 min, followed by reperfusion on a heating blanket. After reperfusion, phosphate-buffered saline (PBS, vehicle), control MSCs, or IFN-γ MSCs (5 × 10^5^ cells/rat) were injected through the abdominal aorta clamped above and below the left renal artery bifurcation. At 7 or 21 days post-injection, rats were sacrificed and their kidneys were collected to evaluate inflammation and fibrosis.

### Immunohistochemistry analysis

Immunohistochemical staining was performed according to previously described methods^47^ using the following primary antibodies: mouse monoclonal anti-Foxp3 (Abcam, Cambridge, UK), rabbit polyclonal anti-CD3 (Dako, Glostrup, Denmark), mouse monoclonal anti-rat CD68 (Serotec, Oxford, UK), and rabbit polyclonal anti-collagen type I (Abcam). FOXP3-, CD3- and CD68-positive cells, as well as areas positive for α-SMA and collagen type I staining, were assessed using ImageJ software (version 1.53 s, NIH) by examining five randomly selected fields (100× magnification) of the cortex.

### Immunohistochemistry analysis (double immunostaining)

Double immunostaining was performed according to the following methods. Sections of formalin-fixed, paraffin-embedded tissues (4 μm thick) were de-paraffinized, subjected to heat-mediated antigen retrieval in citric acid buffer at 98 °C for 40 min, and then blocked in 5% skim milk at room temperature for 1 h. They were incubated with anti-FOXP3 antibody (Abcam) overnight at 4 °C, followed by incubation with the appropriate secondary antibody (DAKO) at room temperature for 1 h, and then incubated with 3,3′-diaminobenzidine (Sigma-Aldrich) at room temperature for 5 min. After that, they were heated again in EDTA buffer (pH 9.0) in the same way. They were then blocked in 2.5% normal horse serum (ImmPRESS Horse Anti-Rabbit IgG Polymer kit; Vector Laboratories, Riverside, CA, USA) at room temperature for 20 min, followed by incubation with anti-CD3 antibody (Abcam) overnight at 4 °C. They were incubated with the secondary antibody (ImmPRESS Horse Anti-Rabbit IgG Polymer kit; Vector Laboratories) at room temperature for 30 min and then incubated with working solution prepared with Vector SG Peroxidase (HRP) Substrate Kit (Vector Laboratories) at room temperature for 5 min.

### Histological analysis

Sections of formalin-fixed, paraffin-embedded tissues (2 μm thick) were stained with Masson’s trichrome to assess fibrosis. Areas of interstitial fibrosis were assessed using Lumina Vision (Mitani, Osaka, Japan) by examining five randomly selected fields (100× magnification) of the cortex.

### Western blot analysis

Sample collection and western blotting were performed as previously reported^36,47^ with the following primary antibodies: anti-VEGFA antibody (Abcam), mouse monoclonal anti-α-SMA (Sigma-Aldrich), rabbit monoclonal anti-TGF-β1 (Abcam), IDO1 polyclonal antibody (Proteintech, Rosemont, IL, USA), mouse monoclonal anti-Foxp3 (Abcam), rabbit polyclonal anti-CD4 (Abcam), and mouse monoclonal anti-GAPDH (Sigma-Aldrich). Horseradish peroxidase-conjugated goat anti-rabbit immunoglobulin G (Dako) or goat anti-mouse immunoglobulin G (Dako) were used as secondary antibodies. SuperSignal West Dura or Pico Systems (Thermo Fisher Scientific, Waltham, MA, USA) were used to detect signals. The intensity of each band was analyzed by ImageJ software and standardized to the level of GAPDH.

### Preparation of conditioned medium

To generate conditioned medium (CM) from untreated MSCs (control MSCs-CM) and IFN-γ MSCs (IFN-γ MSCs-CM), human MSCs (3 × 10^5^ cells/dish) were seeded in 10-cm dishes and cultured in DMEM containing 10% FBS. When the cells reached at least 70% confluence, the medium was replaced with fresh medium with or without 200 ng/mL recombinant human IFN-γ (PeproTech). After 48 h, the culture medium was replaced with DMEM containing 0.1% FBS, which was collected after 48 h.

### Quantitative real-time polymerase chain reaction (qRT-PCR)

RNA extraction and real-time reverse-transcription PCR were conducted according to previously described methods^47^. Specific primers and probes for human IDO1 (assay ID: Hs00984148_m1), and human β-actin (assay ID: Hs99999903_m1) were obtained as TaqMan Gene Expression Assays (Applied Biosystems, Foster City, CA, USA). mRNA levels were normalized to the level of β-actin.

### Enzyme-linked immunosorbent assay (ELISA)

ELISA analysis of IDO (R&D Systems, Minneapolis, MN, USA) was performed according to the manufacturer’s protocol. Concentrations were normalized to the total protein content.

### Isolation of human naïve CD4 T cells

Human peripheral blood mononuclear cells (PBMCs; Biosciences, Berkeley, CA, USA) were suspended with the buffer formulated as MACS® BSA Stock Solution (Miltenyi, Bergisch Gladbach, NRW, Germany) and autoMACS® Rinsing Solution (Miltenyi). Cells were labelled with a Naïve CD4 + T Cell Isolation Kit II (Miltenyi) according to the manufacturer’s protocols. Naïve CD4-positive T cells were sorted by negative selection using LS columns (Miltenyi) and MidiMACS™ (Miltenyi), and then collected.

### Regulatory T cell induction

Naïve CD4 T cells (1 × 10^6^ cells/mL) were cultured in RPMI-1640 (Solarbio, Beijing, China) plus 0.1% FBS (Thermo Fisher Scientific) with MSCs-CM or IFN-γ MSCs-CM at a RPMI-1640:CM ratio of 1:1. Next, Dynabeads human T cell activator CD3/CD28 (Thermo Fisher Scientific) was added at a bead:cell ratio of 1:1, along with animal-free human recombinant IL-2 (ProteinTech) at a concentration of 300 IU/mL, and cells were incubated in a humidified CO_2_ incubator. The medium, IL-2, and beads were exchanged on day 3, and then cells were collected on day 5.

### Transfection of IDO1 siRNA

MSCs were transfected with 20 nM siRNA against IDO1 (s7426, Applied Biosystems) or negative control siRNA (4390843, Applied Biosystems) using Lipofectamine 2000 Transfection Reagent (Thermo Fisher Scientific). After 24 h, transfected cells were washed and fresh complete medium was added. When cells reached 80% confluence, they were collected and subject to in vivo experiments.

### Statistical analysis

Results are expressed as the mean ± standard deviations (S.D.). For multiple group comparisons, one-way ANOVA followed by Bonferroni’s post-hoc test was applied. Comparisons between two groups were analyzed by Student’s t-test. *P* < 0.05 was considered statistically significant.

### Ethical approval and consent to participate

All experimental procedures were approved by the Institutional Animal Care and Use Committee of Hiroshima University (Permit Nos. A15-66 and A17-75).

### Supplementary Information

Supplementary Figure S1.

## Data Availability

The data that support the findings of this study are available from the corresponding author upon reasonable request.
