# Supplementary material for: Mesenchymal stem cells pretreated with interferon-gamma attenuate renal fibrosis by enhancing regulatory T cell induction
Source: Sci Rep. 2024 May 4;14:10251. doi: 10.1038/s41598-024-60928-4 (PMC11069572; doi:10.1038/s41598-024-60928-4)
Supplement: Supplementary file 1 — Supplementary Figure S1. [file 41598_2024_60928_MOESM1_ESM.docx]

**Supplemental material**

**Mesenchymal stem cells pretreated with interferon-gamma attenuate renal fibrosis by enhancing regulatory T cell induction**

So Kurawaki^1^, Ayumu Nakashima^1,2*^, Naoki Ishiuchi^1,2^, Ryo Kanai^1^, Satoshi Maeda^2,3^, Kensuke Sasaki^1^, Takao Masaki^1*^

^1^Department of Nephrology, Hiroshima University Hospital, 1-2-3 Kasumi, Minami-ku, Hiroshima, Hiroshima 734-8551, Japan

^2^Department of Stem Cell Biology and Medicine, Graduate School of Biomedical & Health Sciences, Hiroshima University, 1-2-3 Kasumi, Minami-ku, Hiroshima, Hiroshima 734-8553, Japan

^3^TWOCELLS Company, Limited, 16‑35 Hijiyama‑honmachi, Minami‑ku, Hiroshima, Hiroshima 732‑0816, Japan.

This file includes supplementary figure S1 of western blotting uncropped gel images.


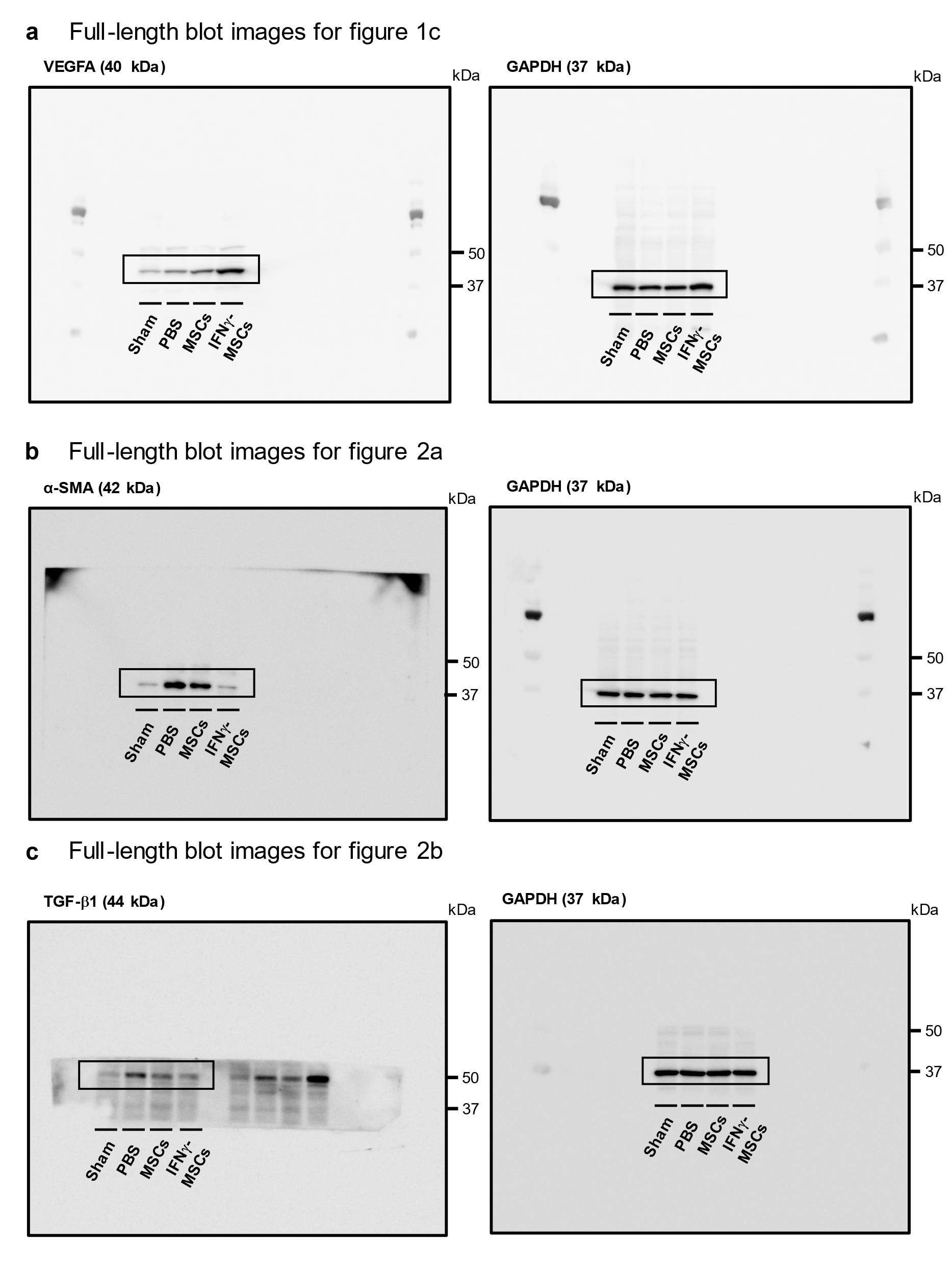


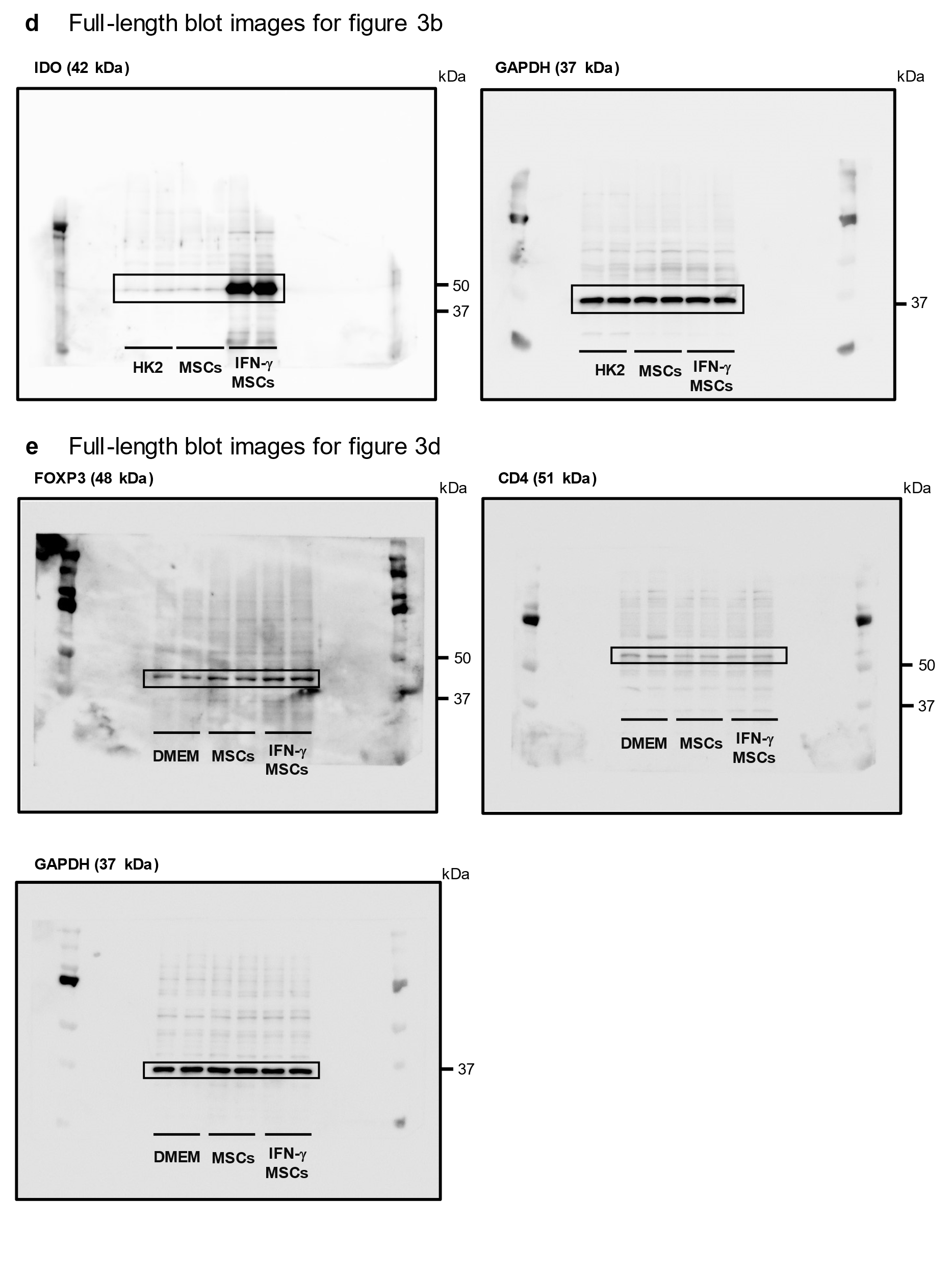


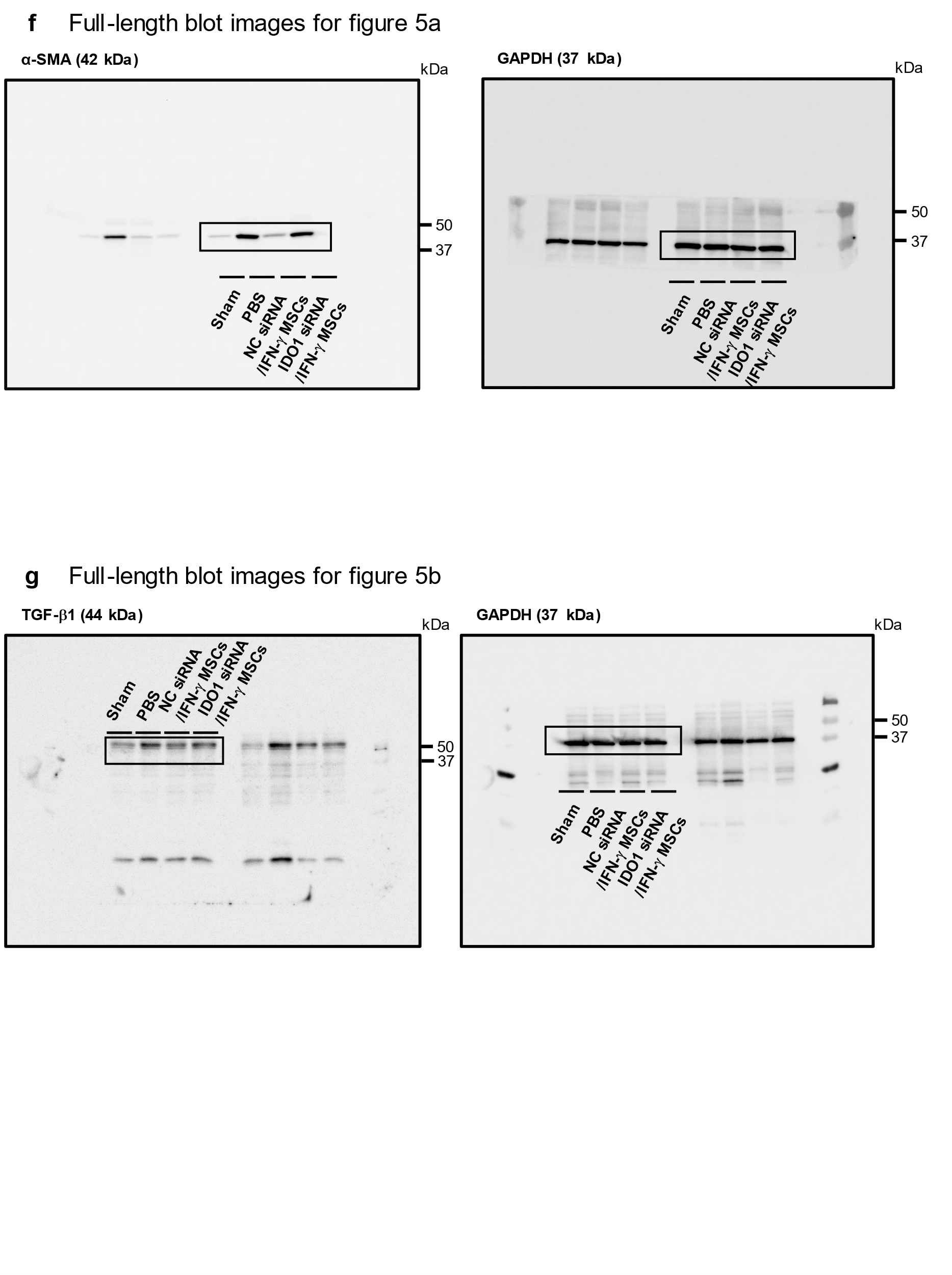


**Western blotting uncropped gel images.** Fig. S1. Full-length western blot images

a Full-length blot images for figure 1c. b Full-length blot images for figure 2a. c Full-length blot images for figure 2b. d Full-length blot images for figure 3b. e Full-length blot images for figure 3d. f Full-length blot images for figure 5a. g Full-length blot images for figure 5b.
